# Bio-Inspired Strategies for Improving the Selectivity and Sensitivity of Artificial Noses: A Review

**DOI:** 10.3390/s20061803

**Published:** 2020-03-24

**Authors:** Charlotte Hurot, Natale Scaramozzino, Arnaud Buhot, Yanxia Hou

**Affiliations:** 1 University Grenoble Alpes, CEA, CNRS, INAC-SyMMES, 17 Rue des Martyrs, 38000 Grenoble, France; Charlotte.HUROT@cea.fr (C.H.); arnaud.buhot@cea.fr (A.B.); 2 University Grenoble Alpes, CNRS, LIPhy, F-38000 Grenoble, France; natale.scaramozzino@univ-grenoble-alpes.fr

**Keywords:** volatile organic compound, olfaction, electronic nose, artificial nose, biomimicry, sensitivity, selectivity

## Abstract

Artificial noses are broad-spectrum multisensors dedicated to the detection of volatile organic compounds (VOCs). Despite great recent progress, they still suffer from a lack of sensitivity and selectivity. We will review, in a systemic way, the biomimetic strategies for improving these performance criteria, including the design of sensing materials, their immobilization on the sensing surface, the sampling of VOCs, the choice of a transduction method, and the data processing. This reflection could help address new applications in domains where high-performance artificial noses are required such as public security and safety, environment, industry, or healthcare.

## 1. Introduction

Biology is a great source of inspiration for the design of advanced technological devices meeting societal and industrial challenges. In particular, several teams have been working on the development of systems that mimic the sense of smell, which would address many applications, from quality control in the food or cosmetics industry to medical diagnostics. These “artificial noses” aim at the detection and identification of volatile organic compounds (VOCs), which are typically small molecular weight organic molecules with a high vapor pressure at room temperature. They can also be referred to as electronic noses, without necessarily relying on an electronic detection method. Unlike most of the biosensors developed for other applications, artificial noses do not target a specific molecule through a lock-and-key recognition, but rather intend to cover the broadest possible spectrum of VOCs. Finally, artificial noses must be distinguished from artificial tongues. In contrast to the latter dedicated for the analysis of samples in the liquid phase, they strive for the detection of VOCs in the gas phase.

**Figure 1:**
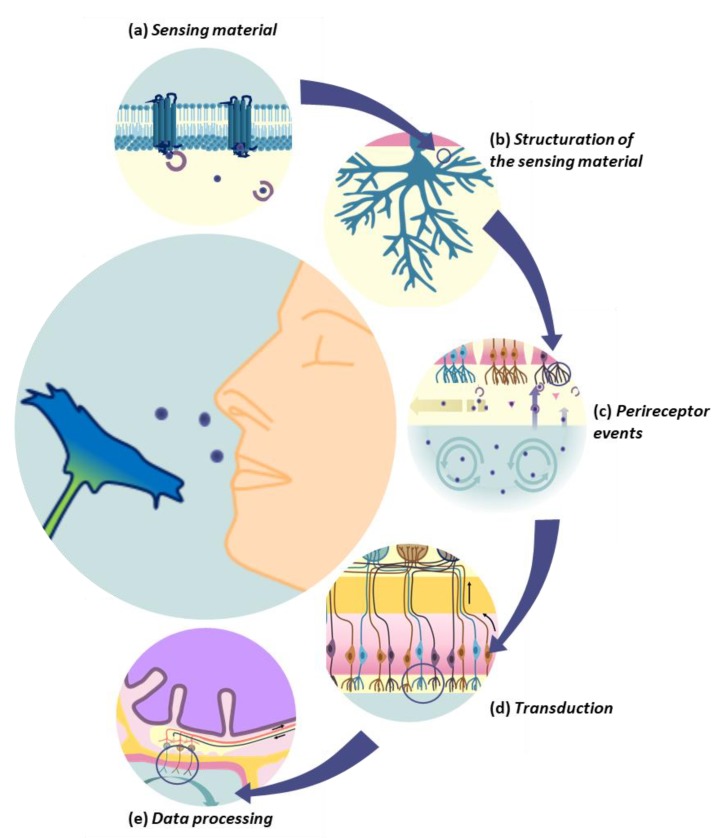
Main constituents and functions involved in the biological olfaction that serve as sources of inspiration for the design of high-performance artificial noses.

To this end, just like their biological counterparts, artificial noses rely on the combination of an array of cross-selective sensing materials and a pattern recognition system [1]. Several different sensing materials are responsible for chemical recognition, a function provided in the biological nose by olfactory receptors (ORs) [2] (Figure 1a). ORs are located on the cilia of olfactory neurons and bath in mucus or lymph (Figure 1b). The VOCs enter the nasal cavity with breathing, and pass through this hydrophilic layer rich in proteins before reaching the receptors (Figure 1c). All the perireceptor events occurring during this step contribute to the detection and identification of VOCs [3]. Olfactory neurons are the transducers of the biological nose (Figure 1d). Their function is to convert the chemical signal resulting from the interaction between a VOC and an OR into an electrical signal that is then transmitted to the olfactory bulb. In the olfactory bulb, several layers of neurons process the signals incoming from all olfactory neurons (Figure 1e). Eventually, the brain analyses the data as a pattern and identify the smell. All the scales of organization of this olfactory system are great sources of inspiration to improve the performances of artificial noses, as we will demonstrate in this review.

Indeed, despite a great number of advances in the last forty years, the performances of the artificial noses developed to date are still far from those of the biological nose. In particular, when it comes to sensitivity and selectivity. For many applications, artificial noses must enable the identification of a target VOC, in a complex milieu, with certainty. Therefore, selectivity is a key feature. It qualifies the ability to detect a target analyte in a sample containing other admixtures and contaminants [4]. Besides, in several medical applications, an artificial nose is required to trace sub-ppm (parts-per-million) concentrations of VOC [5]. The minimum amount of analyte detectable with a sensor defines its limit of detection or sensitivity.

We will review here the technical solutions proposed to improve the sensitivity and selectivity of artificial noses, covering various aspects including the nature of the sensing material used, their structure, their complementarity with the transduction system, as well as the data processing. This paper differs from the numerous reviews in the field, most of which focus on only one of these aspects, through a more systemic approach. This review does not intend to give specific information on the design and working principle of artificial noses, but rather intends to provide suggestions for improving the performance of existing systems. Those relying on metal oxide semiconductors (MOS) or conducting polymers have been extensively studied and reviewed [6], as were devices relying on molecular imprinted polymers [7]. Furthermore, it seems increasingly clear that the use of biological materials and biomolecules as sensitive elements is necessary to approach the performance of the biological nose in terms of odor detection and identification. Nevertheless, so far, systems using whole animals, organs, tissues, or cells are very complicated to industrialize, mainly limited by their stabilities [8,9]. For these reasons, we will consider sensors based on proteins, peptides, DNA, aptamers, and enzymes when it comes to sensing material. However, for every aspect, other than the sensing material, bio-inspired technological solutions applied, so far, only to other kind of sensors will be considered. 

## 2. Design of Biomimetic Sensing Materials

**Figure 2:**
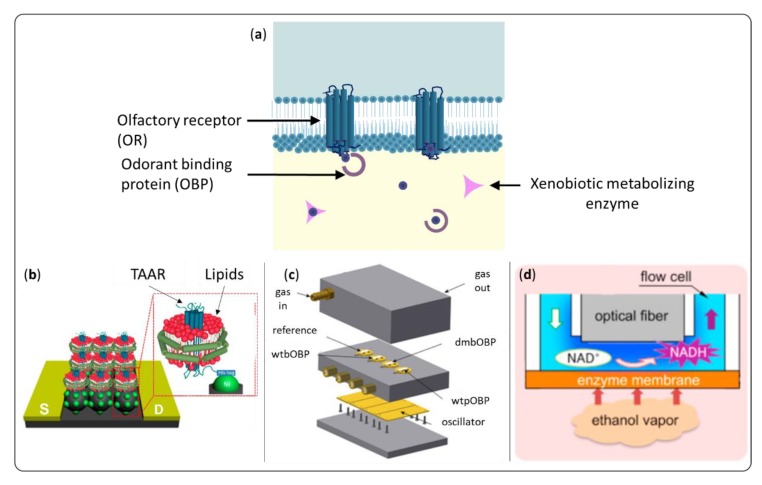
Proteins interacting with volatile organic compounds (VOCs) in the human nose (**a**) and examples of olfactory sensors designed from biosourced materials (**b**). Trace amine associated receptors (TAAR) in lipidic nanodiscs grafted on a field-effect transistor for the detection of cadaverine (adapted from [15]). (**c**) Three different kind of odorant binding proteins (OBPs) deposited on surface acoustic wave sensors for carvone sensing (adapted from [16]). (**d**) Optical sensor for ethanol based on an alcohol dehydrogenase membrane [17].

The deorphanization of human olfactory receptors helped to understand how our nose is able to sense tens of thousands of odorant compounds while being highly discriminating. In fact, some of the ORs are “generalist”, whereas the others are “specialist” and strictly directed toward a limited number of structurally related VOCs [1,10]. It can also happen that a given receptor presents both a high specificity for a molecular feature and cross-reactivity for others [11]. Relying on this observation, the design of narrowly tuned sensing material appears to be the first logical step towards an increase in selectivity. As in the biological nose, these specific materials could be coupled with more “generalist” ones to maintain a wide detection spectrum. Here, we focus on the main strategies that can be adopted to reach this goal (Figure 2). More detailed reviews are available that list the sensors developed so far with this approach [8,12,13].

### 2.1. Bio-Sourced Materials

The use of proteins from the olfactory system as sensing material is the strategy that directly results from this reasoning. Indeed, the biological nose is able to detect some VOCs at parts-per-trillion (ppt) concentration [2]. Studies suggest that it could even have a lower detection threshold than gas chromatography coupled to mass spectroscopy (GC/MS) for given VOCs [14]. To date, the detection performances of three main families of proteins, presented on Figure 2, were investigated. 

ORs are the first candidate that come to mind, by direct mimicry of the olfactory system. Indeed, when successfully immobilized on a sensor, they can greatly improve the selectivity and sensitivity to target VOCs [18,19]. Nevertheless, they are proteins from the G protein-coupled receptor family, composed of seven transmembrane alpha helices, which causes real technological challenges. First, their structure is essential for their binding function, and a lipidic environment is necessary to preserve it. Membrane fractions [20,21], then nanovesicles or nanoliposomes [22,23,24,25], and eventually nanodiscs (Figure 2b) [15,26,27] have been used to achieve their immobilization on sensors. Second, the fact that they are not soluble makes their production challenging. However, high throughput expression of ORs in heterologous cells [28,29,30,31] and cell-free [32,33] strategies exist now. 

Odorant binding proteins (OBPs) are shuttle proteins for the transport of hydrophobic VOCs through the aqueous mucus for vertebrates, or the sensillum lymph for insects. They are smaller than ORs (approximately 20 kDa) and much more stable to temperature change, pH variations, and organic solvents. Moreover, they are soluble, which greatly facilitates their expression by bacteria and their use in sensors [34,35,36]. Mammalian OBPs belong to the lipocalin family. They bind VOCs with affinities in the micromolar range, with relatively low specificity. However, it is possible to tune their binding properties by site-directed mutagenesis [37]. Insect OBPs have a more rigid structure composed of six α-helices stabilized with three disulfide bridges [38]. OBPs’ function requires humidity, and most of the olfactory sensors developed with these proteins to date perform detection in liquid. However, some results show that their use in artificial noses may be possible (Figure 2c) [16,39,40,41,42,43].

To date, very few sensor designs used enzymes for the detection of volatile organic compounds. However, these proteins have a proven high specificity and selectivity, and offer the advantage of not requiring regeneration. One could think in particular of olfactory xenobiotic metabolizing enzymes (XMEs), which are present in the olfactory endothelium and very likely to bind to VOCs [34]. This direction may be worth exploring. Arakawa et al. [17] recently developed gas-phase ethanol biosensors using alcohol dehydrogenase as shown in Figure 2d. Other liquid-based enzyme sensors have been reported for flavor detection [44,45], or water quality determination [46].

### 2.2. Biomaterials by Computational Design

Although promising, the industrial application of bioelectronic noses relying on proteins is still very challenging. The immobilization of these molecules on the sensor surface is difficult to perform without a loss of their structure and activity, and in a reproducible manner. The computational design of high affinity peptides could help to overcome these limitations [47]. Peptides are much more robust than proteins, cheaper to synthesize, and could potentially be integrated into industrial devices. On top of that, they can reach similar affinity to those of proteins through rational design. Several methods commonly used for drug discovery could be adapted for this purpose, including molecular modeling and molecular docking, quantitative structure-activity relationship, de novo design, virtual screening, and molecular dynamics simulation [48].

#### 2.2.1. From Proteins to High-Affinity Peptides

**Figure 3:**
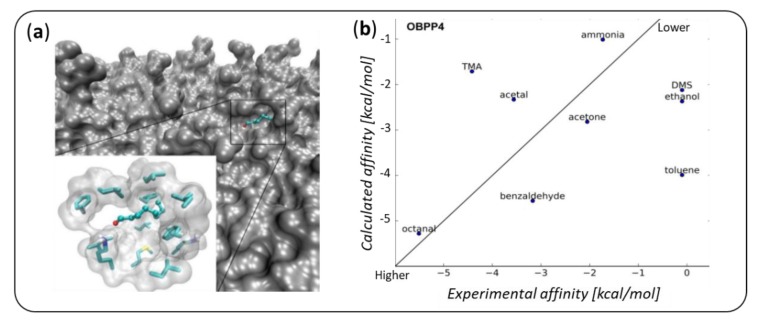
(**a**) Docking simulations performed on an OBP 3D structure model enable to identify a binding site for octanal for obtaining specific peptides accordingly. (**b**) The experimental and calculated affinities are consistent and show that one of the peptides has a high affinity for octanal [57].

First, several teams have been trying to extract the sequences responsible for VOC recognition in ORs [49,50,51,52,53], OBPs [54,55,56,57], or antibodies [58]. As an example, presented in Figure 3, authors [57] used docking simulations on an OBP to extract peptides selective for octanal, and tested the influence of the chain length on the selectivity. In accordance with experimental results obtained with a piezoelectric sensor, the longest peptide mimicking the binding pocket of the protein was the best candidate. Knowledge of the three-dimensional structure of these proteins is a staple for a successful simulation. Class A G-protein-coupled receptors of known structures provide a pattern to compensate for the lack of high-resolution 3D structures of ORs in most cases [59]. Docking simulations are then carried out on the obtained molecular model.

#### 2.2.2. Virtual Screening 

The previous approach can be limited when there is no natural ligand inventoried for a target molecule. In this context, virtual screening represents an interesting alternative. Blanco et al. [60] paved the way to molecular modeling of peptides binding to VOCs. Later, Pizzoni et al. [61] designed five peptides with various physicochemical properties and predicted their binding scores with 14 VOCs from different chemical classes. The comparison with experimental quartz crystal microbalance (QCM) gas sensing proved that the forecasts were accurate. DNA can also be used for the design of gas sensors [62,63,64], with sequence dependent selectivity [65]. Recently, Mascini et al. [66] reported the use of hairpin DNA screened in silico against different chemical classes of VOCs. Their use on QCM and surface plasmon resonance (SPR) imaging chips was successful for gas sensing [67].

### 2.3. Novel Materials Based on High-Throughput Selection Methods

Natural evolution provided us with a method to carry out this screening process in vitro [68]. Several screening methods inspired by this mechanism made it possible to identify specific molecular binders from a library, experimentally [69]. Among them, phage display and SELEX (Systematic Evolution of Ligands by Exponential Enrichment) represent promising tools for the design of peptides and proteins or oligonucleotides (respectively) with a high affinity and specificity toward a target VOC. 

#### 2.3.1. Phage Display

Phage display is a laboratory technique that enables the selection of peptides or proteins with a high affinity for a target [68]. It uses bacteriophages, viruses that infect bacteria. In this technique, a random genetic sequence is inserted into a phage coat protein gene, causing the phage to “display” a random peptide or protein on its envelope. It results in a connection between genotype (the gene) and phenotype (the peptide or protein). A whole library of phages, each displaying a different peptide is constituted. The target, here a VOC, is immobilized on a surface, which is put in contact with the phage library. In this way, the phages that display a peptide with a high affinity for the target will bind to the surface. After incubation, the unbound viruses are rinsed off, while the bounded ones are eluted and amplified through the infection of bacteria. As a result, an enriched library of phages with a high affinity for the target is obtained. This library is used for the next selection round. Commonly, three–five rounds are carried out, at the end of which the strongest binders are retrieved, isolated, and their genetic material sequenced. Thus, the corresponding peptide sequence can be traced back.

Although phage display has been widely used to discover binders for large molecules [70,71], the literature is scarcer for smaller targets. Interesting examples are the detection of explosives [72,73,74,75] and pesticides [76,77]. Indeed, the screening of peptides selective to a VOC is particularly challenging, since it requires immobilizing the odorant molecule on a surface. Besides, the affinity between a short peptide and a VOC is relatively low. Still, Sawada et al. [78] identified a naphthalene-specific peptide from a commercial library of M13 phages exhibiting 12-mer peptides. It exhibits good sensitivity and selectivity in solution, but the team did not test the detection in the gas phase. 

**Figure 4:**
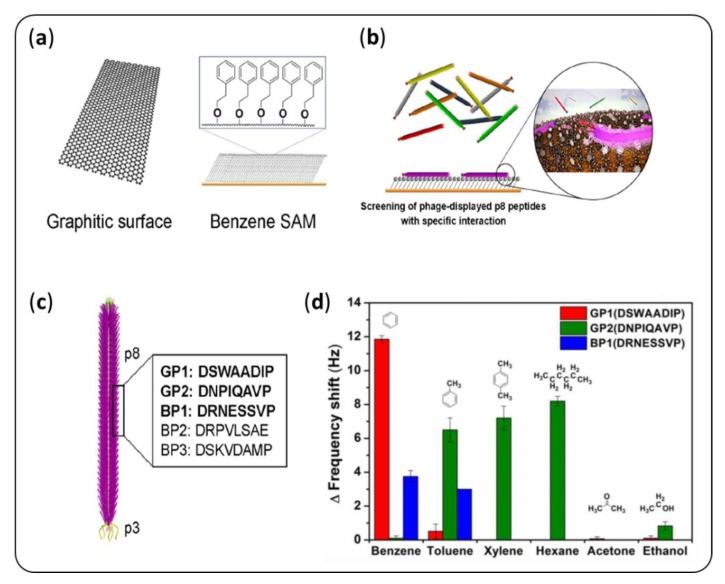
Example of a phage display protocol proposed by Ju et al. [79]. A library of M13 phages with random peptides displayed on the p8 coat protein was screened (**b**) against a graphene surface or a benzene SAM (**a**) to identify ligands specific to benzene. The selected peptides (**c**) allow for single-carbon discrimination (**d**).

The use of peptides or proteins from the phage display for gas detection represents an additional challenge, since the selection is carried out in solution. Ju et al. [79] screened a phage library against benzene, as depicted in Figure 4. Their results are very promising: the three selected peptides showed high selectivity for benzene, even in the gas phase. To achieve such a performance, they chose to work with a custom phage library with p8 proteins displaying random peptides [80]. This made selection possible, even with weaker affinities [81]. However, Tanaka et al. [82] recently managed to perform the highly sensitive detection of benzaldehyde in solution and in the gas phase with a peptide selected from a seven-mer commercial library. Nonetheless, they did not clearly demonstrate the selectivity of this peptide. 

The phage display protocol would therefore require additional adaptations to be feasible on small targets. The selection of VOC-specific proteins by this method would be worth exploring [83]. It is noteworthy that Nakamura et al. [84] proposed an on-bead selection to identify pentapeptides binding selectively to dioxin. This could represent an interesting cell-free alternative to phage display.

#### 2.3.2. SELEX

Speaking of cell-free screening methods, SELEX is probably the most common one. It allows the selection of nucleic acid aptamers, which have the combined advantages of being cheap, robust, and reusable, and can have high sensitivity and specificity to a target [85]. New sensing strategies rely on their conformational changes upon target binding. 

The selection process begins with the synthesis of a very large oligonucleotide library consisting of randomly generated sequences of fixed length flanked by constant 5′ and 3′ ends that serve as primers [86]. Just like in phage display, the library is incubated with the target. Oligonucleotides with a weak affinity to it are removed. The bound sequences are eluted and amplified by polymerase chain reaction (PCR) to prepare for subsequent rounds of selection. After a few cycles, the best binders are sequenced. The detection of small molecules, and so of VOCs is made easier by the use of Capture-SELEX, which enables the selection of DNA aptamers for solute targets [87]. In this method, the same docking sequence is added to all the oligonucleotides, to enable their immobilization on magnetic beads bearing the complementary strand. Then during selection, oligonucleotides with high affinity to the target will preferentially bind to it, and so be released from the beads. The weak binders are easy to remove with a magnet. 

Nevertheless, to date, all of the aptamers developed for VOC sensing were only tested in the liquid phase. Examples include the work of Komarova et al. [88] for the detection of furaneol and Kuznetsov et al. [89] for the detection of vanillin. In both papers, the selected aptamers were coupled with an ion-sensitive field-effect transistor. The latter integrated an air-to-liquid interface (see Section 4.2).

In summary, there are many strategies to design sensing materials with a high affinity to a target, which can be very helpful to design sensors dedicated to a given application. These high-affinity materials can be integrated in an artificial nose, in association with more “generalist” ones to keep a wide spectrum of detection. Nevertheless, we have to keep in mind that increasing the affinity between a probe and a target will ineluctably make the regeneration and the reuse of the sensor more difficult [90]. Even in the biological nose, the signal termination is a complex problem. Enzymes, proteins and a continuous renewal of the mucus and the cells are probably involved in this mechanism [3,34]. For this reason, it is worth combining this strategy with a systemic biomimetic design to increase the selectivity and sensitivity of a sensor, which we will discuss in the next parts.

## 3. Immobilization of the Sensing Material on the Sensor Surface

**Figure 5:**
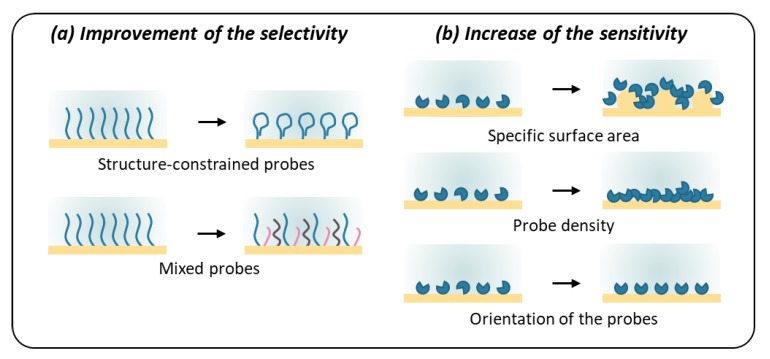
The immobilization of the sensing material on the surface of the sensor is key to make full use of its sensing potential and to ensure the highest sensitivity and selectivity.

Once the probes selected, their arrangement and structuration upon immobilization on the sensor surface is crucial. In this part, we will review the functionalization strategies that lead to improved sensitivity and selectivity of an artificial nose (Figure 5).

### 3.1. Improvement of the Selectivity

At the molecular level to begin with, ORs and OBPs are highly structured, which ensures their activity. When switching to synthetic probes, a similar structure mimicking “binding pockets” of these proteins is most likely desirable to increase specificity. The fact that most of the peptides selected by phage display to date exhibit a secondary structure reinforces this hypothesis. Indeed, in the work of Sawada et al. [78], circular dichroism spectroscopy showed that the selected peptide folds in β-turn. For Tanaka et al. [82], the best binders included a proline, which can contribute to a bent or twisted conformation. In both cases, the authors considered the structuration of the peptides was partly responsible for the molecular recognition. 

Giebel et al. [91] were the first to voluntarily constrain the conformation of peptides by flanking them with two cysteine which would result in disulfide bond formation. The cyclic peptides selected by phage display had a three-order-of-magnitude higher affinity to streptavidin than linear ones. Moreover, they are on the rise for drug development [92]. Structures mimicking antibodies are also emerging in drug discovery [93,94], which could be very interesting for the selection or maturation of peptides by phage display. Since a secondary structure is only possible for primary sequences of more than four–five amino acids, the length of the peptides could become a key feature when it comes to the improvement of the selectivity [57,95].

**Figure 6:**
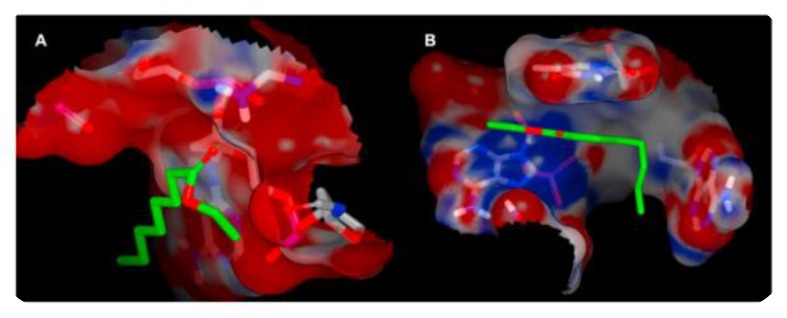
Electrostatic molecular surfaces of the ssDNA CTGCAA, with a planar interaction surface (binding score –2.26 k/mol) (**A**), and ATAATC with a saddle shaped binding pocket (binding score –6.28 k/mol) (**B**) in complex with ethyl octanoate (highlighted in green) [66].

Mascini et al. [66] used the same reasoning to develop hairpin-DNA probes whose loops recreate structured “binding sites”. In silico simulations, in Figure 6, showed that saddle-shaped binding pockets lead to higher binding scores than planar ones, which the team explained by a higher synergic cooperation. 

Interestingly, this idea can also be useful to improve a natural binder. Kotlowski et al. [96] produced mutants of the Italian honeybee OBP 14 with an additional disulfide bond, reducing its dynamics and leading to a higher affinity for eugenol. 

The team of Dr. Y. Hou [97,98,99,100] proposed another approach to improve the selectivity of artificial tongues, which could be adapted to artificial noses. It relies on mimicking the biological properties of glycosaminoglycans based on a combinatorial approach. Two disaccharides, lactose and sulfated lactose, were used as building blocks, mixed in different proportions and self-assembled on the sensor so as to reproduce the charged topography of these polysaccharides. In a similar way, peptides could be mixed to fabricate “binding-pocket like” surfaces in a cheap and easy to produce way. 

### 3.2. Increase of the Sensitivity

**Figure 7:**
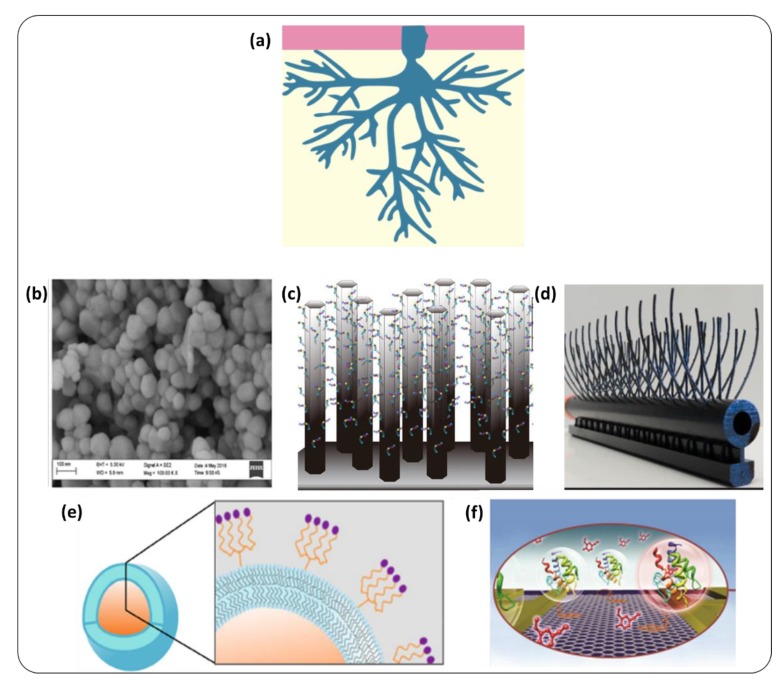
(**a**) Olfactory neurons deploy a large specific surface area in the mucus through their cilia. To mimic this strategy, (**b**) Mascini et al. [103] modified ZnO nanoparticles with peptides, (**c**) Tanaka et al. [82] immobilized peptides specific to benzaldehyde on ZnO nanowires. (**d**) Jaffar-Bandjee et al. [108] fabricated artificial “moth antennae” (**e**) Gray et al. [109] proposed liposomes displaying tetrameric peptides to enable cooperative binding (**f**) Kotlowski et al. [96] used a bio-functional linker to optimize the orientation of OBPs on a graphitic surface.

At the tissue level, the structure of the olfactory epithelium plays a key role in the sensitivity of the biological nose [101]. For instance, in humans, ten million olfactory neurons project five to fifty cilia each into the mucus (schematic representation in Figure 7a). As a result, the total sensing area can reach about 70 cm² [95]. Similarly, in artificial sensors, a nano-structuration of the sensing surface leads to an increase in the sensitivity. As a first example, the team of Dr. D. Compagnone [66,102,103] deposited functionalized nanoparticles on their sensors to increase the specific surface, as shown in Figure 7b. Zine et al. [104] generated them directly in situ. Recently, Tanaka et al. [82] immobilized selective peptides on ZnO nanowires, Figure 7b. 

Similarly, the shape of insect antennae extends a very large sensitive surface. Besides, this configuration is adapted to the diffusion of VOCs in air. For example, a molecule encountering the antenna of *Bombyx mori* would hit and bounce on its surface about 100 times [105]. In such a way, it acts as a physical amplifier [106], which could be a great source of inspiration for sensor design. Spencer et al. [107] mirrored the structure of moth antennae thanks to an additive fabrication process. This system developed for fundamental understanding purposes could inspire technological devices, as pointed in the review of Jaffar-Bandjee et al. [108] (Figure 7d).

The density of sensing molecules on the sensor surface is also a very important point when it comes to the sensitivity. With an optimal density, the sensitivity can be improved. Moreover, multivalency is indeed a proven approach to improve affinity between a probe and a target [110,111]. This is particularly relevant for peptides selected by phage display. When isolated, they may actually exhibit considerably lower affinity than when presented on the phages, where they can attach multivalently to targets. For example, dendritic architectures inspired from the phage structure (Figure 7e) can typically improve the affinity by two orders of magnitude [109,112]. Alternatively, Hou et al. [40] used a Langmuir–Blodgett technique to create a dense layer of OBPs. 

Besides the strict number of probes per surface unit discussed above, their orientation is also key to ensure an optimized binding, especially when it comes to proteins. In the literature, various ingenious strategies have been developed for this purpose. OBPs can be immobilized using a cysteine [37] or histidine tag [96,113] at their N-terminus so as to keep a good access of the VOCs to the binding pocket. Kotlowski et al. [114], Larisika et al. [114], and Zhang et al. [115] proposed a solution with a bi-functional linker (Figure 7f). Du et al. [116] employed aptamers to graft olfactory receptors onto their sensor. Interestingly, Kuang et al. [117] used a graphene-binding peptide as an anchor.

## 4. Strategies Inspired by the Perireceptor Events

**Figure 8:**
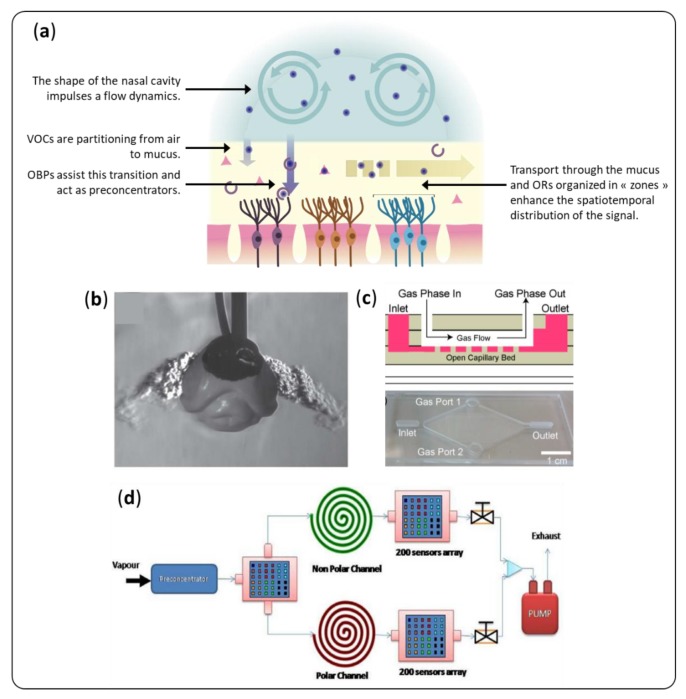
(**a**) Main perireceptor events in the biological olfaction. (**b**) 3D printed model of a dog nose enabled Staymates et al. [119] to visualize, understand, and reproduce the sniffing process. (**c**) Warden et al. [120] proposed an open channel microfluidic card for gas-to-liquid extraction. (**d**) The design proposed by Harun et al. [121] helped to separate a sample spatially and temporally.

The biological nose is a much more complex system than just an array of cross-selective sensors. Prior to reach olfactory receptors, VOCs have first to enter the nasal cavity through breathing. Then, they pass through an aqueous layer—the nasal mucus in vertebrates or the sensillar lymph in insects. This milieu is constantly renewed and rich in proteins that interact with VOCs. Perireceptor events, described roughly in Figure 8a, include all biochemical interactions that occur during these two stages. They are necessary to ensure the good performances of the natural nose, and they affect the perception of smells. When it comes to artificial noses, the sampling of VOCs plays a crucial role [118]. The perireceptor events become an important source of inspiration for their sensitive and selective detection.

### 4.1. Flow Dynamics

In the biological noses of vertebrates, breathing conveys the VOCs to the olfactory epithelium. The airflow dynamics plays a role in olfaction. First, the shape of the nasal cavity directs most of the flux towards the olfactory epithelium, acting as a pre-concentrator [122]. Furthermore, it distributes spatially the VOCs depending on their physicochemical properties, which facilitates their identification. Sniffing could reinforce this effect [123]. 

Dr. D. R. Walt’s team was the first team to take inspiration from this observation [124]. They improved the discrimination capacity of a fiber optic sensor using a replica of canine nasal cavity in which they placed identical sensors at different positions. The spatial distribution of the VOCs due to flow dynamics in this chamber provided useful supplementary data. Chang et al. [125] adopted a similar strategy and designed a fluidic cell on the model of the human nasal turbinate to facilitate the discrimination of VOCs. A good understanding of the flow dynamics in the analysis chamber can also result in the improvement of the sensitivity of the sensor. Scott et al. [126] used a computational fluid dynamic model to position their QCM sensor in a way to collect most of the sample. It is also a way to limit the effects of position and the response time of the sensors [127]. 

Some teams have even mimed sniffer-induced flow modulations to improve the sensitivity and discrimination capabilities of their sensors. El Barbri et al. [128] used a peristatic pump to modulate the flow of the carrier gas in their artificial nose. It resulted in an improvement of the classification accuracy for five volatile organic compounds. The implementation of this method required dedicated data processing such as discrete wavelet transform or Deep-Q network. The latter, when integrated into a feedback loop, can help to optimize the flow modulation [129]. The breathing frequency is indeed a proven important parameter in artificial sniffing devices [130]. Staymates et al. [119] coupled the two previous approaches using both a simulated 3D printed dog’s nose and sniffing. This system, presented in Figure 8b, resulted in a 16-fold improvement of the sensitivity of a commercially available explosive detector.

### 4.2. Hydrated Sensing Environment

In vertebrates, before reaching the olfactory epithelium, inspired VOCs must first penetrate a mucus layer. It plays an important role in the transport of VOCs, their metabolization, and their interaction with olfactory receptors [3]. Moreover, thanks to the presence of large glycoproteins (mucins), mucus provides a hydrated environment, which is very favorable for fragile proteins. It guarantees the integrity of their structure, and therefore their function. In insects, this function is assured by the sensillum lymph. Similarly, humidity is most probably a key factor for the design of selective and sensitive sensors. 

Although OBPs are more stable than other proteins, they can lose their active conformation in harsh conditions. Thus, an aqueous medium is required to ensure their function [36]. Humidity remains an important issue when it comes to peptides, although less structured and so, more robust than proteins when dried. The water molecules seem to play a role in the specific interaction between peptides and a target VOC. Indeed, the sensitivity and specificity of selected peptides can be lost at low ambient humidity [79,131]. 

To circumvent this problem, several teams have recently elaborated designs by integrating an air-to-liquid extraction interface to an artificial tongue. Lee et al. [132] and Kuznetsov et al. [89] used a porous membrane made in polycarbonate and SiO_2_/Si_3_N_4_/SiO_2_, respectively. This membrane acts similarly to the cuticle in insect antennae. It prevents the evaporation of water while allowing the diffusion of odorant molecules. Warden et al. [120] proposed another strategy with open microfluidic channels relying on capillarity (Figure 8c). Alpha–cyclodextrin added in the capture liquid enabled reasonable capture efficiency, up to 30% for hexanal at 16 ppm.

Although interesting, these approaches pose the problem of the transition of VOCs from a gaseous phase to a liquid phase, which can drastically increase the detection time and the limit of detection. In the biological nose, this limitation turns to an asset: OBPs assist the partitioning of hydrophobic VOCs from air to the mucus, and this acts as a pre-concentration step of the chemical message. In the absence of these carrier proteins, artificial devices integrating an air-to-liquid interface would likely require a first step of pre-concentration of VOCs [133,134].

### 4.3. Chromatographic Effects

Mucus presents another advantage for the selective detection of VOCs. Indeed, molecules travel differently through it, depending on their physicochemical properties, as they would in a chromatographic column. Thereby, it creates a spatial and temporal separation, which greatly facilitates the discrimination of VOCs [135,136,137].

Gas chromatography coupled with mass spectroscopy (GCMS) is a reference technique for analyzing VOCs in industry. It is very reliable and sensitive with a limit of detection below the part per billion. Moreover, it can provide the exact composition of a complex mixture, component by component and sometimes in a quantitative way. The analysis of samples for food quality, health, or environment monitoring is made complex by: (1) the wide range of masses of the constituents of these complex mixtures and (2) the chemical resolution needed to distinguish between two VOCs with very similar structure. Nevertheless, the development of multidimensional gas chromatography (MDGC) provides a partial solution to these problems [138]. It is based on the series connection of several columns with stationary phases of orthogonal properties. Notably, heart-cut MDGC is particularly adapted to the analysis of VOCs in real samples [139]. Another option is to analyze the resulting spectrum, not by identifying the single peaks, but as a signature for the whole mixture. It can be considered as an artificial nose in which the different sensors are chromatographic peaks. Notably alpha-MOS (Toulouse, France) is selling a flash gas chromatography electronic nose, used in many studies [140].

It is also possible to integrate a spatial separation on conventional artificial noses to improve their performance. Mimicking the chromatographic behavior of the mucus, Covington et al. [141] integrated a microfluidic channel coated with a polymer onto an array of chemoresistive microsensors. They noticed improvements in the selectivity of their artificial nose thanks to the temporal retention of VOCs. Later on, the same team [121,142] used a combination of three chemosensor arrays, as drawn in Figure 8d. The first one provided spatial information and separated the sample between two chromatography columns. Like in MDGC, one column was coated with a polar stationary phase, and the other with a non-polar one. These columns ensured temporal separation of the samples, and conveyed them to two other sensor arrays. The system gained in its ability to discriminate between complex odors thanks to spatiotemporal response. In both designs, the polymer used to mimic mucus was unrelated with real molecules present in the nose. It could be interesting to put these works in perspective with Yabuki et al. [143], who studied the separation of VOCs through a chromatography column coated with OBPs.

However, adding these elements (water and artificial mucus) on the chip is not without risk. A poor design could lead to a dramatic increase of the non-specific adsorption. Woodka et al. [144] ingenuously designed a low volume analysis chamber combined with a low vapor flow to separate the VOCs directly by adsorption on the sensors, without adding a stationary phase and obtained very good discrimination results as well. Another option is to consider strategies to block non-sensitive surfaces beforehand.

## 5. Transduction Systems

**Figure 9:**
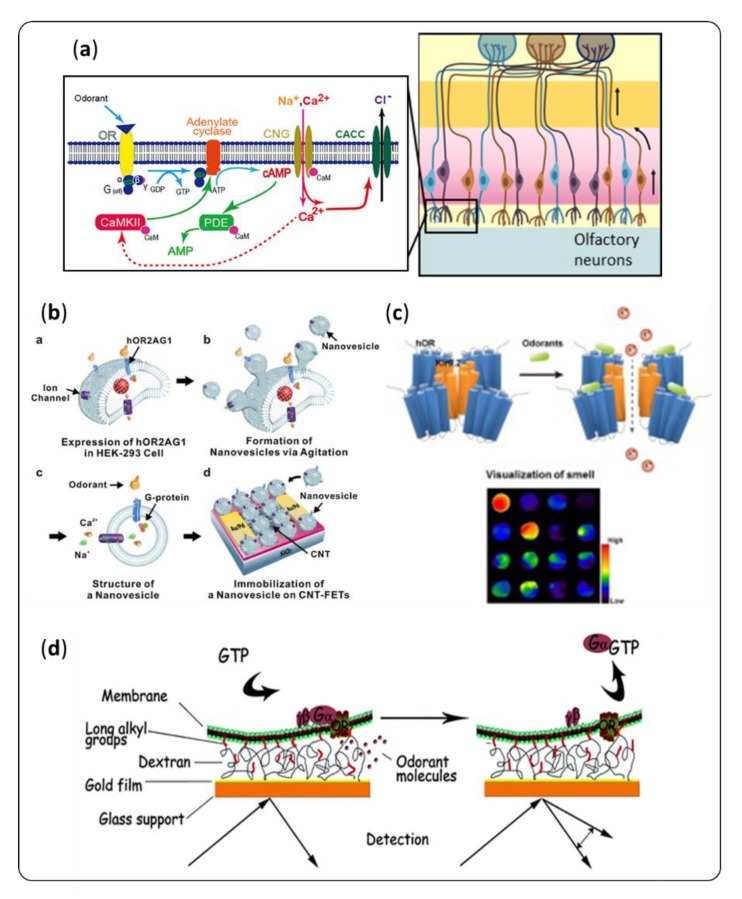
(**a**) The olfactory neurons are the biological transducers of the olfactory signal. On the left is a schematic representation of the 3, 5-cyclic monophosphate (cAMP) signaling pathway [148]. This process inspired different designs to achieve highly sensitive detection of VOCs. (**b**) Jin et al. [23] integrated nanovesicles containing ORs and ion channels on field–effect transistors to enhance the potential difference caused by VOC binding. (**c**) Oh et al. [149] designed ion channel-coupled ORs for fundamental research purpose that could be integrated in biosensors. (**d**) The design proposed by Vidic et al. [22] used the desorption of the G protein upon VOC binding to enhance an SPR signal.

Once a VOC molecule finally reaches the olfactory epithelium and interacts with an OR, the olfactory neuron responds with an electrical output signal. Different transduction pathways may coexist [145,146]. One example is the adenosine 3, 5-cyclic monophosphate (cAMP) signaling (Figure 9a). Briefly, the activation of the OR, coupled to a G protein and adenylyl cyclase results in the production of cAMP, a second messenger. cAMP opens Ca^2+^ ion channels, creating a current, which is amplified by Ca^2+^-gated Cl^–^ channels. Some cell-based olfactory sensors exploit this complex mechanism [147]. However, “industrializable” artificial noses must rely on transduction methods that are easier to manufacture, more stable over time and better controlled. Classical methods can be mechanical (QCM, microcantilever, surface acoustic wave, etc.), electronic (pellistor, field effect transistor (FET), etc.) or optical (SPR, absorbance, fluorescence, etc.). They will not be detailed in this review, which will focus on biomimetic strategies to improve their sensitivity and selectivity.

### 5.1. Bioinspired Amplification and Transduction

The binding of a VOC molecule to a sensor causes a very weak raw electrical, mechanical or optical variation, and so it is interesting to consider adding an amplification system to the design. This is where olfactory neurons come into play as sources of inspiration [150]. 

It is possible to enhance the measurable potential difference due to the recognition of a VOC molecule by an OR using ion channels, just like in the biological nose. Jin et al. [23] immobilized nanovesicles containing both ORs and ion-channels on a field-effect transistor (Figure 9b). They obtained a sensitivity two orders of magnitude higher than that of a similar bioelectronic nose without calcium ion channels. Oh et al. [149] coupled human ORs with an ion-channel to monitor the binding of odorant with a membrane potential dye (Figure 9c). This technique was developed for more fundamental research purposes, but these ion-channel coupled receptors could be used in biosensors.

As far as optical transducers are concerned, when binding an odorant, some proteins can undergo a change in their optical properties that can act as an amplifier. As a first example, OBPs conformation can change [151,152]. This event can lead to a variation in their refractive index [153], which Hurot et al. [37] exploited to amplify an SPR signal. Secondly, tryptophan and tyrosine fluoresce when excited at a correct wavelength. This phenomenon can quench in the close vicinity of a VOC. Thus, if one of those residues is located in the binding pocket of a receptor, it can act as a binding reporter. This is the case for most natural insect OBPs [36]. Based on this principle, Wei et al. [154] designed a specific mutant of porcine OBP adding a tryptophan residue in its binding pocket. This engineered protein could be used in a biosensor, using direct fluorescence measurements. Returning to the primary inspiration of olfactory neurons, Vidic et al. [22] co-expressed an OR and a G protein in a yeast, and used some membrane extract on an SPR sensor (Figure 9**d**). When an odorant binds to the receptor, the G subunit is desorbed, leading to a much bigger change in refractive index. In 2008, the same team immobilized ORs-containing nanosomes on an SPR chip. Those receptors bound specifically a kind of OBP. Upon injection of the receptor preferential VOC, the OBPs were released, providing the same amplification effect on the SPR signal [155]. 

It is interesting to note that, in the two latter cases, an important variation in mass accompanied the change in optical properties. It could be adapted to mechanical transducers. It is also the case for aptamers: Andrianova et al. [156] designed an aptamer, which releases its complementary DNA strand during VOC binding. They used an ion sensitive field effect transistor as a transducer, but similar probes could also be interesting for amplifying optical or mechanical detection.

### 5.2. Multiplexing for Large Arrays of Sensors

However, designing the best transducers is not enough to optimize the sensitivity and selectivity of an artificial nose. To mimic the function of the biological nose, it is necessary to multiplex them in order to obtain the largest possible array of sensors, in term of both variety and number of sensors [157]. Indeed, humans express about 400 different types of functional ORs [1]. One olfactory neuron carries about one million replicates of the same kind, with the olfactory epithelium being composed of 10 million olfactory neurons. This layout has several advantages. First, the variety of binding properties among the ORs increases the range of perceptible VOCs. Second, a greater number of chemical “pixels” allows a finer pattern and therefore better identification of these compounds. Thirdly, a large number of replicates increases drastically the signal to noise ratio while smoothing out signals from defective receptors. Finally, it allows to take full advantage of the spatial and temporal separation allowed by an artificial mucus or a fluidic chamber (see Section 4). This last point also requires a transduction method that provides kinetic signals.

**Figure 10:**
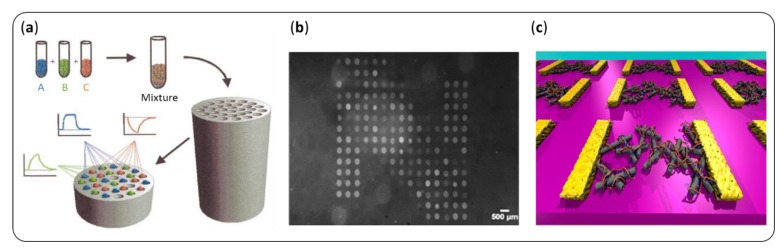
Multiplexing the sensors is key to achieve high sensitivity and selectivity of an artificial nose. (**a**) Dickinson et al. [159] proposed a fiber optic bead sensor array. (**b**) Brenet et al. [161] constructed a microarray and used surface plasmon resonance imaging (SPRi) for VOC sensing in the gas phase. (**c**) Kybert et al. [63] integrated numerous graphene field effect transistors (FETs) on a small chip.

Concerning the choice of a transduction method, care must be taken to ensure that it does not induce interference and distortion between signals from different sensors [158]. In this respect, optical transduction methods are particularly suitable for the development of sensitive and reliable artificial noses. Moreover, contrary to multiplexed electronic transducers or mechanical resonators, some of them offer the possibility of versatile, small scale, and repeatable functionalization with biomolecules. For instance, since 1999, the Walt group [159] developed a fiber optic bead-based sensor with thousands of microspheres in a very easy to produce way (Figure 10a). These sensors provide spectral and temporal responses and are suitable for on-site analysis. A review for all other microbead sensors is available [160]. Brenet et al. [161] used SPRi to monitor simultaneously four replicates of 18 different sensing materials deposited in a microarray format (Figure 10b). A spotting robot achieved the functionalization of the sensing surface simply, via thiol chemistry. Again, this transduction method provides a label-free, kinetic response. Even more simple, colorimetric and fluorometric sensors are gaining attention [162].

Multiplexing electrical transducers may be more complex, especially for wiring reasons, and because the resulting sensors will be independent from each other, making their comparison less straightforward. Nevertheless, nanotechnology is a key ally to design highly sensitive olfactory sensors [163,164]. Indeed, nanoscale materials combine their high conductivity with a high surface-to-volume ratio, which allows the dense functionalization of their surface with biomolecules (see Section 3.2). As a result, the most sensitive olfactory sensors developed to date relied on an electronic transduction, and measured a change in current, conductivity, or electric potential in a biomolecule upon the binding of a VOC. Moreover, nanofabrication holds out hope for progress in scalability. For instance, Kybert et al. [64] developed a photolithography method to integrate 112 graphene-based FETs with good performances on a single chip with the size of a coin (Figure 10c). It is then easy to graft biomolecules onto graphene by means of π–π stacking. In this paper, DNA strands permitted the selective detection of VOCs in vapor. For the record, Larisika et al. [114] attached OBPs on the same kind of device, for the detection of VOCs in solution. Carbon nanotubes are commonly used for the development of olfactory sensors with an enhanced sensitivity [165]. They were notably decorated with olfactory receptors [27] or specific peptides [56,117]. On top of that, the development of micro-fabrication techniques made these sensors more scalable [166]. As a final example, Gao et al. [167] designed a highly sensitive olfactory sensor with silicon nanowires conjugated with OBPs. Its fabrication relied on a top–down approach, which is much cheaper and easier to implement in a large scale than the classical bottom-up strategy.

## 6. Data Processing

The interaction of a sample with all the sensors of a large array will give a vector of high dimension that we will call “signature”. The aim is then to identify the sample from its signature. First of all, the signatures of a large number of known samples are collected in a database and used for the training phase. Later on, when an unknown sample is analyzed, its signature is compared with the database to identify it. Multidimensional analysis methods are particularly suited to large data, above all when the difference between samples can be subtle. These methods reduce the number of dimensions to be observed in a way to best represent the variability of the data. Principal component analysis (PCA) and linear discriminant analysis (LDA) are classically used. Some reviews [168,169] provided a detailed description of the classical pattern recognition methods implemented in artificial olfaction.

Here, we focus on the biomimetic data processing strategies used to improve the sensitivity and selectivity of artificial noses [157,170,171]. Most of the work presented below was performed on MOS or conductive polymer artificial noses, but the same computing principles could be used for bioelectronic systems.

### 6.1. A Model of Neurons: Toward Spike-Based Neuromorphic Approaches

As the front-end of the olfactory pathway, olfactory neurons use time as a dimension for coding [172]. Odor information is transmitted to the higher brain areas in the form of spike trains. The firing rate of olfactory neurons and the variations of this feature encode part of the information about the nature of an odor. Similarly, the temporal component of the signals given by the transducer of an artificial nose conveys additional information that is useful for improving its performance [97]. For instance, Brenet et al. [161] greatly improved the classification of alcohols and carboxylic acids by integrating kinetic descriptors in PCA and hierarchical clustering on principal components. Nevertheless, adding features to the data processing increases the dimensionality of the problem and can lengthen the recognition time. 

Concerning the detection time, it is possible to identify VOCs with a good accuracy using only transient information. Several approaches were considered for arrays of MOS [173,174]. Interestingly, Luo et al. [175] proposed a gradient tree-boosting algorithm that only needed the first 6 s of the raw signals to achieve the classification of six gases. This solution is interesting, since it does not require feature extraction from the data. Rodriguez et al. [176] achieved a similar result with a deep multilayer perceptron neural network to identify wine spoilage in less than three seconds. Munoz-Mata et al. [177] held the same reasoning with QCM sensors. 

To tackle the high dimensionality, it is crucial to select the optimal set of features to cover most of the information. Calculating the correlation between them is a classical method to do so [178,179]. Perera et al. [180] chose a bioinspired technique, which allowed a robust classification, even with a small number of samples. Clusters (signals) from different features (OR types) converged to feature groups (glomeruli), and this redundancy enabled noise reduction and VOC discrimination. This feature selection can also be interesting to get rid of the drift or defective sensors [181,182]. Remarkably, Magna et al. [183] also developed a self-repairing algorithm dedicated to this purpose.

Besides, the reduction of dimensionality itself leads to expensive and power-consuming data processing. The requirement for large computing devices limits the portability of the devices. This leads us to neuromorphic methods, which take the comparison with the functioning of neurons one-step further and code only the necessary information in the form of spikes [170,184]. They combine the advantage of requiring low power, simplifying the data processing, and enabling learning algorithm, as we will see in the next part. 

### 6.2. Artificial Neuron Networks and Hardware Models of Olfactory Bulb

**Figure 11:**
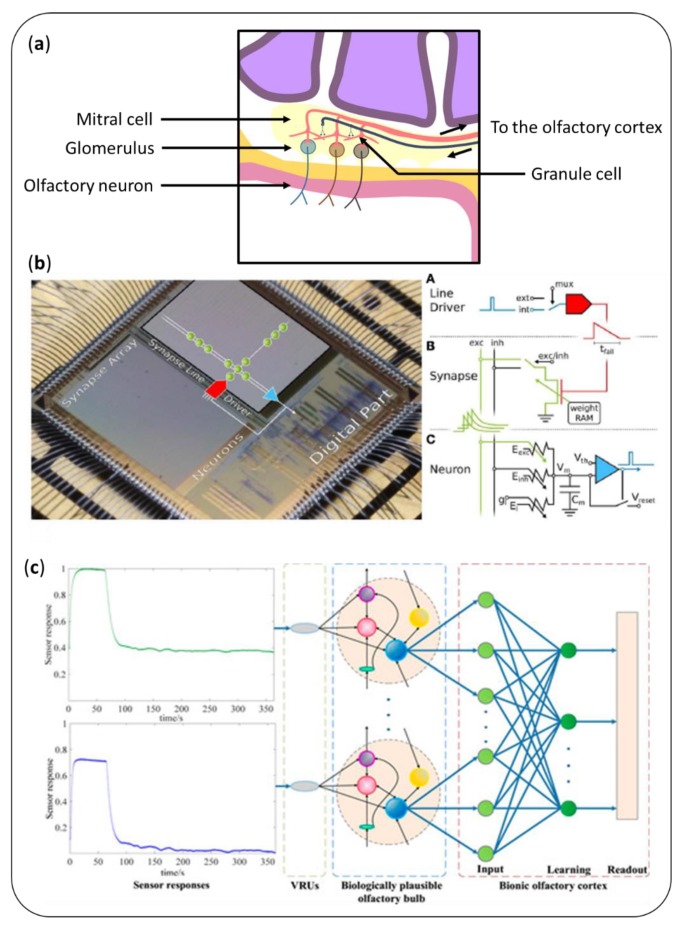
(**a**) Several layers of cells are involved in the biological processing of odor signals. To reproduce their functions, (**b**) Pfeil et al. [195] proposed a hardware model of the olfactory bulb. (**c**) Liu et al. [197] coupled a computational model of the olfactory bulb with one of the olfactory cortex.

In the biological nose of mammals, several layers of neurons are involved in the processing of the signal [185,186]. All the olfactory neurons carrying the same type of olfactory receptors converge on the same spatial area called the glomerulus, where they synapse with mitral cells. Then, mitral cells will send the averaged signal through their axons to the olfactory cortex. The dendrites of mitral cells contact granule cells, which by some theories produces lateral inhibition between mitral cells (Figure 11a). The function of the olfactory bulb is crucial to take advantage of large sensor arrays (see Section 5.2). A very similar structure evolved in insects, called antennal lobe, where projection neurons are the equivalent of mitral cells [187]. Logically, most of the solutions considered for artificially reproducing these structures relied on artificial neuron networks [170].

Initially, several teams carried out computer simulations of the architecture of the olfactory bulb [188,189,190]. Notably, Martinelli et al. [191] described an original work with a colorimetric sensor. With a single layer of spiking neurons, they showed that the latency enables quick discrimination of VOCs. Jing et al. [192] developed a neuron network that mimicked olfactory neurons, mitral cells, and granule cells to classify raw data from an artificial nose with 93% accuracy. 

Interestingly, spike-based neuromorphic approaches are compatible with analog data processing, and so with hardware implementation. Koickal et al. [193] demonstrated the first example of a hardware model of olfactory bulb to process spike signal coming from an olfactory sensor. They implemented a time-dependent learning circuit, which adapted the weights of the model in a dynamic fashion. Besides lowering the power consumption of the data processing unit, it enabled one to get rid of the variations between the different MOS sensors. This was further improved by including onset latency for a better classification [194]. Pfeil et al. [195] introduced supervised learning based on reward-depending plasticity with an accuracy ranging from 87% to 96% (Figure 11b). Recently, Vanarse et al. [196] deployed a spiking neuron network hardware with a 96.5% accuracy in the identification of target gases.

### 6.3. Feedback Inhibitory Loops and Learning Algorithms Taking Inspiration from the Cortex

In the mammalian nose though, the olfactory bulb is only a “secondary transduction” which acts as a signal-conditioning step. The olfactory cortex performs the identification of the odor [186]. Similarly, in insects, the higher brain centers include the mushroom body and the lateral protocerebrum.

Ratton et al. [198] were the first to use a simulation integrating both an olfactory bulb and an olfactory cortex connected by a feedback loop. This model analyses a VOC in a hierarchical fashion. This can be very interesting when it comes to analyzing unknown samples. Still, its linearity resulted in a poor success rate. Subsequently, different teams focused on making their solution suitable for real-world applications. The NEUROCHEM project [199] proposed a very large-scale sensor array (65,536 elements) using conducting polymers. The European universities involved in this project explored different neuromorphic algorithms implemented in a platform suitable for robotic integration. Diamond et al. [200] proposed a model of the insect antennal lobe, able to process noisy signals in real time. Recently, Borthakur et al. [201] utilized an external plexiform layer to enable online training without risk of forgetting and integrating a confidence indicator. Figure 11c shows the design of Liu et al. [197], who used squared cosine receptive fields to improve the classification performance. 

The olfactory cortex is also very useful for the habituation capacity of the nose, through its inhibitory feedback. Granule cells could indeed act as a feedback loop between it and the mitral cells. The solution proposed by Li and Hertz [202] intended to mimic the habituation of the biological nose. In particular, the aim was to identify a variation in a mixture over time. Guttierez-Osuna et al. proposed several other algorithms in his wake, one of them based on the KIII model of the cortex, which is more biologically plausible [203,204].

## 7. Conclusions and Perspectives

Artificial noses developed to date are not yet as sensitive and selective as the biological nose. However, such performances are desirable to address biomedical, industrial, or public safety applications. It is the multi-scale and systemic organization of the biological nose that allows it to reach detection limits in the order of ppt, and to distinguish several hundred thousand odors. This includes special binding properties of olfactory receptors, the appropriate structuring of the olfactory epithelium, relevant peri-receptor events, the unique functioning of neurons and the different neuronal layers involved in data processing. 

To improve the performances of the artificial noses, it is therefore relevant to draw inspiration from all these elements in order to propose ingenious biomimetic solutions, as summarized in this review. 

Several strategies are implemented for the development of more selective sensitive materials, either by using biological proteins or by screening novel probes in vitro or in silico. Besides, the biomolecules under consideration are often fragile. Technical solutions for regeneration, self-healing, or reinforcement must therefore be envisaged to enable the industrialization of the corresponding artificial noses. Moreover, in order to exploit the full potential of sensitive materials, particular care must be taken in their immobilization on the sensor surface, which allows a high sensitivity and selectivity. Indeed, the structuration of the probes, their density and their appropriate orientation are essential. Also, not to be overlooked is the importance of VOC sampling in terms of fluid dynamics and hydration. Finally, as far as data transduction and processing is concerned, the trend is towards large arrays of sensors, which generate a large amount of data. Neuromorphic data processing methods will make it possible to process these data while limiting calculation time and cost. 

However, all of the methods presented above have their limitations, and one cannot expect to match the performance of the biological nose by digging in only one direction. The solution probably lies in a system that optimizes each of the above elements to combine them. As perspectives, in order to get closer to the performance of the biological nose, first, one may combine both “generalist” and “specialist” sensitive materials on the same large sensor array. Indeed, it is interesting to note that high discrimination can be obtained with non-specific probes based on combinatorial approaches, provided they are sufficiently numerous and their physicochemical properties varied enough [161,205]. For targeted applications however, the development of specific materials by the methods developed above could allow the confident detection of particular VOCs even in complex environments. Second, such a large sensor array could be immersed in an artificial mucus and then integrated into an analysis chamber with optimal configuration for the fluid dynamics. Despite some progress in this field, inspired by peri-receptor events, very little work has sought to replicate an artificial mucus that is truly biomimetic, as has been done in other fields [206]. In particular, the pre-concentration effect that exists in the biological nose during the partition of VOCs in this aqueous phase has no artificial counterpart yet. Third, relying on the observation that a flavor is usually perceived from the combined effect of olfaction and taste, some research groups have been working with a combination of an electronic nose and tongue [207]. This strategy has been successful for improving the identification capabilities of the sensors and could be promising for the next generation of artificial noses. Finally, clearly, the improvement of the performances of the artificial nose will benefit from the rise of artificial intelligence. The choice of a strategy will of course have to be made according to the targeted applications.
